# Supplementary material for: End Sequence Analysis Toolkit (ESAT) expands the extractable information from single-cell RNA-seq data
Source: Genome Res. 2016 Oct;26(10):1397–410. doi: 10.1101/gr.207902.116 (PMC5052061; doi:10.1101/gr.207902.116)
Supplement: Supplemental Material [file supp_gr.207902.116_Supp_Materials.tar.gz › ESAT-esat_v0.1/doc/html/classbroad_1_1core_1_1math_1_1_distribution.html]

ESAT: broad.core.math.Distribution Class Reference


ESAT


- Main Page
- Related Pages
- Packages
- Classes
- Files

- Class List
- Class Index
- Class Hierarchy
- Class Members

All Classes Namespaces Files Functions Variables Enumerator Pages

- broad
- core
- math
- Distribution

Static Public Member Functions |
List of all members

broad.core.math.Distribution Class Reference

|  |  |
| --- | --- |
| Static Public Member Functions | |
| static double | noncentralChiSquareDensity (double x, double nu, double lambda) |
|  | |
| static double | noncentralChiSquareCdf (double x, double nu, double lambda) |
|  | |
| static double | skellamDensity (int k, double lambda1, double lambda2) |
|  | |
| static double | skellamCdf (int k, double lambda1, double lambda2) |
|  | |
| static double | skellamPvalue (int k, double lambda1, double lambda2) |
|  | |
| static double | skellamLeftTail (int k, double lambda1, double lambda2) |
|  | |
| static double | skellamRightTail (int k, double lambda1, double lambda2) |
|  | |

## Detailed Description

Author
:   prussell Statistical distributions

## Member Function Documentation

|  |  |  |  |
| --- | --- | --- | --- |
| static double broad.core.math.Distribution.noncentralChiSquareCdf | ( | double | *x*, |
|  |  | double | *nu*, |
|  |  | double | *lambda* |
|  | ) |  |  |

static

Compute the cdf of a noncentral chi square distribution

Parameters
:   |  |  |
    | --- | --- |
    | x | Value of random variable |
    | nu | Degrees of freedom |
    | lambda | Noncentrality parameter |

Returns
:   The cdf of the noncentral chi square distribution with the specified parameters, evaluated at the specified value

Here is the caller graph for this function:

|  |  |  |  |
| --- | --- | --- | --- |
| static double broad.core.math.Distribution.noncentralChiSquareDensity | ( | double | *x*, |
|  |  | double | *nu*, |
|  |  | double | *lambda* |
|  | ) |  |  |

static

Compute the pdf of a noncentral chi square distribution

Parameters
:   |  |  |
    | --- | --- |
    | x | Value of random variable |
    | nu | Degrees of freedom |
    | lambda | Noncentrality parameter |

Returns
:   The pdf of the noncentral chi square distribution with the specified parameters, evaluated at the specified value

Here is the caller graph for this function:

|  |  |  |  |
| --- | --- | --- | --- |
| static double broad.core.math.Distribution.skellamCdf | ( | int | *k*, |
|  |  | double | *lambda1*, |
|  |  | double | *lambda2* |
|  | ) |  |  |

static

Compute the cdf of a Skellam distribution, the distribution of the difference of two Poisson random variables

Parameters
:   |  |  |
    | --- | --- |
    | k | Value of random variable |
    | lambda1 | Poisson parameter for distribution 1 |
    | lambda2 | Poisson parameter for distribution 2 |

Returns
:   Probability that the difference (variable1 minus variable2) is less than or equal to the specified value

Here is the call graph for this function:

Here is the caller graph for this function:

|  |  |  |  |
| --- | --- | --- | --- |
| static double broad.core.math.Distribution.skellamDensity | ( | int | *k*, |
|  |  | double | *lambda1*, |
|  |  | double | *lambda2* |
|  | ) |  |  |

static

Compute the pdf of a Skellam distribution, the distribution of the difference of two Poisson random variables

Parameters
:   |  |  |
    | --- | --- |
    | k | Value of random variable |
    | lambda1 | Poisson parameter for distribution 1 |
    | lambda2 | Poisson parameter for distribution 2 |

Returns
:   Probability that the difference (variable1 minus variable2) is equal to the specified value

Here is the call graph for this function:

|  |  |  |  |
| --- | --- | --- | --- |
| static double broad.core.math.Distribution.skellamLeftTail | ( | int | *k*, |
|  |  | double | *lambda1*, |
|  |  | double | *lambda2* |
|  | ) |  |  |

static

Compute the left tail of a Skellam distribution, the distribution of the difference of two Poisson random variables

Parameters
:   |  |  |
    | --- | --- |
    | k | Value of random variable |
    | lambda1 | Poisson parameter for distribution 1 |
    | lambda2 | Poisson parameter for distribution 2 |

Returns
:   The probability of observing at most this value under the null hypothesis of a Skellam-distributed random variable

Here is the call graph for this function:

Here is the caller graph for this function:

|  |  |  |  |
| --- | --- | --- | --- |
| static double broad.core.math.Distribution.skellamPvalue | ( | int | *k*, |
|  |  | double | *lambda1*, |
|  |  | double | *lambda2* |
|  | ) |  |  |

static

Compute the one-tailed P-value of a Skellam distribution, the distribution of the difference of two Poisson random variables

Parameters
:   |  |  |
    | --- | --- |
    | k | Value of random variable |
    | lambda1 | Poisson parameter for distribution 1 |
    | lambda2 | Poisson parameter for distribution 2 |

Returns
:   The probability of observing at least as extreme a value under the null hypothesis of a Skellam-distributed random variable (whichever tail is smaller)

Here is the call graph for this function:

|  |  |  |  |
| --- | --- | --- | --- |
| static double broad.core.math.Distribution.skellamRightTail | ( | int | *k*, |
|  |  | double | *lambda1*, |
|  |  | double | *lambda2* |
|  | ) |  |  |

static

Compute the right tail of a Skellam distribution, the distribution of the difference of two Poisson random variables

Parameters
:   |  |  |
    | --- | --- |
    | k | Value of random variable |
    | lambda1 | Poisson parameter for distribution 1 |
    | lambda2 | Poisson parameter for distribution 2 |

Returns
:   The probability of observing at least this value under the null hypothesis of a Skellam-distributed random variable

Here is the call graph for this function:

Here is the caller graph for this function:

---

The documentation for this class was generated from the following file:

- java/broad/core/math/Distribution.java


---

Generated on Fri Aug 15 2014 15:10:52 for ESAT by  

 1.8.7
